# The impact of advertising on women’s self-perception: a systematic review

**DOI:** 10.3389/fpsyg.2024.1430079

**Published:** 2025-01-07

**Authors:** Yao Dai, Zhixuan Zhu, Wu Yuan Guo

**Affiliations:** ^1^Faculty of Social Sciences, University of Macau, Taipa, Macao SAR, China; ^2^Faculty of Arts and Humanities, King’s College London, London, United Kingdom; ^3^Department of Curriculum and Instruction, The Education University of Hong Kong, Tai Po, Hong Kong SAR, China

**Keywords:** advertising, women, self-perception, body image, gender roles, female psychology

## Abstract

This systematic review explores the multifaceted psychological impact of advertising on women’s self-perception, examining traditional advertising, femvertising, and the emerging effects of digital transformation. By synthesizing evidence from 95 peer-reviewed studies, this review examines the relationship between media portrayals of women and key psychological outcomes, including body image, self-esteem, self-objectification, and gender role attitudes. Our analysis reveals that traditional advertising, which often features idealized and stereotypical portrayals of femininity, continues to be associated with negative psychological outcomes such as increased body dissatisfaction and self-objectification. These effects appear to be intensifying in digital contexts, where exposure is more frequent and pervasive. Femvertising—advertising that aims to challenge gender stereotypes and empower women—shows promise in promoting positive psychological outcomes, but its effectiveness is contingent on perceived authenticity and individual viewer characteristics. The digital transformation of advertising has created new dynamics in how women encounter and process advertising messages, with social media platforms enabling both new forms of feminist messaging and new risks of superficial appropriation. Our findings suggest that understanding advertising’s impact requires consideration of platform-specific features, user characteristics, and content types. The review highlights the need for advertising practices that prioritize genuine representation and empowerment of women, while also addressing the unique challenges and opportunities presented by digital platforms. This work contributes to a deeper psychological understanding of advertising’s impact on women’s self-perception and calls for updated theoretical frameworks and practical approaches that can address both traditional and emerging forms of advertising exposure.

## 1 Introduction

### 1.1 Background

Advertising is a ubiquitous presence in modern society, with individuals exposed to hundreds, if not thousands, of advertisements each day (Kilbourne, 1999; Story, 2007). As a powerful force in shaping societal norms and individual perceptions, advertising has the potential to influence how individuals view themselves and others from a psychological perspective (Pollay, 1986; Schroeder and Borgerson, 1998). This is particularly true for women, who have long been the target of advertising messages that promote narrow and often unrealistic standards of beauty, femininity, and gender roles (Belkaoui and Belkaoui, 1976; Courtney and Whipple, 1983; Goffman, 1979).

The psychological impact of advertising on women’s self-perception has been a topic of concern and investigation for decades (Kilbourne, 1994; Lavine et al., 1999). Research has consistently shown that exposure to idealized images of women in advertising can lead to negative psychological outcomes, such as increased body dissatisfaction, self-objectification, and internalization of the thin ideal (Bessenoff, 2006; Dittmar and Howard, 2004; Grabe et al., 2008). These findings are particularly troubling given the pervasive nature of advertising and the increasing pressure on women to conform to narrow beauty standards (Wolf, 1991; Fredrickson and Roberts, 1997). Moreover, traditional advertising has been criticized for its limited and often stereotypical portrayals of women, which can reinforce traditional gender roles and sexist attitudes from a psychological standpoint (Cortese, 2015; Furnham and Mak, 1999; Matthes et al., 2016). Women in advertisements are frequently depicted as homemakers, caregivers, or sexual objects, with their psychological value tied primarily to their appearance and adherence to gender norms (Courtney and Whipple, 1983; Goffman, 1979). These representations can have a detrimental impact on women’s self-perception, as they promote a narrow view of what it means to be a woman and can lead to the internalization of gender stereotypes (Lavine et al., 1999; MacKay and Covell, 1997).

In the past few years, there has been an increasing acknowledgment of the necessity for broader and more empowering portrayals of women in advertising (Akestam et al., 2017; Drake, 2017). This trend has given rise to “femvertising,” a term coined to depict advertising that defies conventional gender stereotypes and aims to empower women through affirming and inclusive communication (Becker-Herby, 2016; Champlin et al., 2019). Femvertising campaigns, such as Dove’s “Real Beauty” and Always’ “Like a Girl,” have garnered widespread attention and praise for their efforts to promote body positivity, self-confidence, and gender equality (Bissell and Rask, 2010; Taylor et al., 2016). However, despite the promising potential of femvertising, its psychological effectiveness in promoting positive self-perception among women remains a topic of debate and investigation (Abitbol and Sternadori, 2016; Kapoor and Munjal, 2019). Some scholars have argued that femvertising, while well-intentioned, may still operate within the confines of consumer culture and reinforce the notion that women’s empowerment can be achieved through consumption (Gill and Elias, 2014; Johnston and Taylor, 2008). Others have questioned the authenticity of femvertising messages and the extent to which they reflect genuine commitments to gender equality (Vredenburg et al., 2020).

As society evolves in gender topics and the research on gender deepens, it seems that women begin to play more diversified and powerful roles in society, which are progressively reflected in advertising industry. In addition to traditional female-related ads and femvertising, some advertising campaigns such as #MoreWomen, #Timesup, and #MeToo aimed to encourage females to fight against abuse and get involved in politics, which in ads reflects portrayals of female social power by mostly focusing on female consumers and being conveyed through female models’ agentic power (Kordrostami and Laczniak, 2022; Tsai et al., 2021). Some feminist studies (Yoder and Kahn, 1992) have noticed that female power is portrayed in ads into “power-over” (e.g., an individual’s power and control over other people or over the environment) and “power-to” (an individual’s control over their own thoughts, behaviors, and feelings). Varghese and Kumar (2020) found there are increasing advertising messages to show characteristic representations of women, women’s talents, and pro-women messages due to various factors like activism and regulatory efforts in ads. Similarly, scholars also concluded that the ad environment is changing in a way that ads containing femvertising-based messages are being more featured (Akestam et al., 2017; Windels et al., 2020). Eisend (2010) further pointed out that more research on new gender-role attributes is needed in advertisements as such attributes are traditionally perceived with one gender but have recently been employed by both males and females in ads. The present study partially answered this by investigating how consumers perceive power as an attribute that can be associated with women in ads. All these findings draw future research attention to the increasing number of ads that diversified features of females’ exercising power in recent years in addition to traditional female-related ads and femvertising. Thus, Kordrostami and Laczniak (2022) timely concluded that “it is important to understand how these different dimensions might interact with each other” and “how different segments of consumers (gender, age, education) respond to different portrayals of female power”(p. 1203). They stressed the necessity of future research in investigating females’ perception of the simultaneous application of two or more power domains and sources of female power such as female models’ age and authenticity in ads.

Recent research has revealed increasingly sophisticated dynamics in how digital media platforms shape women’s self-perception and responses to gendered advertising. Studies examining TikTok beauty content have found that even brief exposure can trigger immediate negative psychological effects, including increased appearance shame, anxiety, and lowered self-compassion (Seekis and Kennedy, 2023). However, the relationship between content and impact appears more nuanced than simple exposure effects. Analysis of successful female-oriented TikTok accounts indicates that authenticity, emotional connection, and entertainment value are more important for engagement than pure beauty content (Du et al., 2022).

The evolution of feminist advertising approaches has also grown more complex. Recent research identifies six key dimensions that shape perceptions of authentic “femvertising”: transparency, consistency, identification, diversity, respect, and challenging stereotypes (Hainneville et al., 2023). This framework helps distinguish genuine feminist advertising from “femwashing”–superficial appropriation of feminist themes without authentic commitment. Importantly, consumers appear increasingly sophisticated in evaluating advertising authenticity, with the ability to identify nuanced differences between performative and genuine feminist messaging.

This emerging research points to several critical developments: (1) The psychological impacts of beauty content remain powerful but are increasingly mediated by authenticity and connection; (2) Digital platforms enable new forms of feminist messaging but also new risks of superficial appropriation; and (3) Consumers have developed more sophisticated frameworks for evaluating gendered advertising claims. These findings suggest the need for more nuanced theoretical models that can account for both the persistent power of beauty ideals and emerging forms of resistance and reinterpretation.

### 1.2 Rationale and objectives

The need for a comprehensive review of advertising’s impact on women’s self-perception has become increasingly urgent for several compelling reasons. First, the rapid evolution of advertising platforms and formats, particularly the rise of social media and influencer marketing, has fundamentally changed how women interact with advertising messages (Garcia et al., 2022; Tiggemann and Anderberg, 2020). These changes have created new dynamics in how advertising affects women’s psychological well-being, yet our understanding of these effects remains fragmented. Second, while femvertising has emerged as a significant trend in advertising, its effectiveness in promoting positive self-perception among women remains debated, with some studies suggesting potential limitations and unintended consequences (Abitbol and Sternadori, 2019; Åkestam et al., 2021). Third, the simultaneous presence of both traditional advertising and femvertising creates a complex media environment whose cumulative psychological impact on women requires systematic investigation.

Several critical gaps in the existing literature underscore the importance of this review. While previous reviews have examined specific aspects of advertising’s impact, such as body image (Grabe et al., 2008; Want, 2009) or the effectiveness of femvertising (Abitbol and Sternadori, 2016; Champlin et al., 2019), no systematic review has comprehensively synthesized the full range of psychological effects across different advertising approaches in the current media landscape. This gap limits our understanding of how various forms of advertising collectively influence women’s self-perception. Additionally, despite the coexistence of traditional advertising and femvertising, little research has systematically examined how these different approaches interact to shape women’s psychological well-being. Current literature also lacks a thorough examination of how individual differences and cultural factors moderate advertising’s impact on women’s self-perception, hampering our ability to develop targeted interventions and recommendations. Furthermore, the psychological impact of advertising on newer platforms, particularly social media and influencer marketing, remains understudied compared to traditional advertising formats - a significant gap given the increasing prominence of these platforms in women’s daily media consumption.

Based on these gaps and motivations, this systematic review aims to achieve four specific objectives. First, we seek to synthesize and evaluate current evidence regarding advertising’s psychological impact on women’s self-perception across different platforms and formats. This includes examining the effects of traditional advertising in the modern media landscape, the impact of emerging advertising formats, and the cumulative influence of diverse advertising approaches. Second, we aim to critically assess the effectiveness and limitations of femvertising by analyzing its success in promoting positive self-perception, identifying potential unintended consequences, examining its interaction with traditional advertising messages, and evaluating its authenticity in different contexts. Third, we intend to identify and analyze key factors that moderate advertising’s impact on women’s self-perception, including individual psychological characteristics, cultural factors, media literacy levels, and platform-specific effects. Fourth, the transition from traditional to digital advertising platforms has created new dynamics in how advertising affects women’s self-perception, yet our understanding of these technological effects remains limited. While recent studies have begun examining platform-specific impacts (e.g., Seekis and Kennedy, 2023), we need a comprehensive understanding of how advertising effects have evolved with new technologies.

By addressing these objectives, this review aims to study how different forms of advertising influence women’s self-perception in the contemporary media environment. This understanding is crucial for developing more effective and responsible advertising practices, informing policy decisions, and identifying strategies to promote positive psychological outcomes for women exposed to advertising messages.

### 1.3 Theoretical framework

The impact of advertising on women’s self-perception can be understood through the lens of several theoretical perspectives, which provide a framework for examining the psychological and social processes involved in this relationship. Two key theories that have been widely applied in this context are objectification theory (Fredrickson and Roberts, 1997) and social comparison theory (Festinger, 1954).

Objectification theory, as posited by Fredrickson and Roberts (1997), suggests that women in Western cultures often experience objectification, where their worth is predominantly tied to their physical appearance. This objectification can occur through various forms of media, including advertising, which often presents women as sexual objects or focuses disproportionately on their bodies (Kilbourne, 1999; Schroeder and Borgerson, 1998). As per objectification theory, frequent exposure to such visuals can prompt women to adopt an external viewpoint regarding their own bodies, termed as self-objectification (Fredrickson and Roberts, 1997; Keltner et al., 2003). This phenomenon has been associated with various adverse psychological effects, such as body shame, appearance anxiety, and eating disorders (Calogero, 2004; Harper and Tiggemann, 2008; Moradi and Huang, 2008). This theory offers valuable insight into how the objectifying portrayal prevalent in traditional advertising may fuel women’s negative self-perception and body image issues.

Social comparison theory, developed by Festinger (1954), posits that individuals inherently assess themselves by comparing their skills and qualities to those of others. In advertising, this theory elucidates how exposure to idealized depictions of women can foster unfavorable self-assessments and dissatisfaction with one’s body (Bessenoff, 2006; Tiggemann and McGill, 2004). According to social comparison theory, when women view advertisements featuring thin, attractive models, they may engage in upward social comparisons, perceiving themselves as falling short of the idealized standard (Richins, 1991; Tiggemann and Polivy, 2010). These comparisons can lead to increased body dissatisfaction, as well as other negative outcomes such as lowered self-esteem and negative affect (Bessenoff, 2006; Tiggemann and McGill, 2004). Moreover, social comparison theory suggests that the negative impact of idealized images may be particularly pronounced for women who are more prone to engaging in social comparisons or who have pre-existing body image concerns (Dittmar and Howard, 2004; Tiggemann and Polivy, 2010). This highlights the importance of considering individual differences in susceptibility to the negative effects of advertising on self-perception. Table 1 summarizes the key tenets and implications of objectification theory and social comparison theory for understanding the impact of advertising on women’s self-perception.

**Table 1:** Theoretical perspectives on the impact of advertising on women’s self-perception.

Theory	Key tenets	Implications for advertising and self-perception
Objectification theory (Fredrickson and Roberts, 1997)	- Women are frequently objectified and valued primarily for their appearance; Exposure to objectifying media can lead to self-objectification.	- Traditional advertising often objectifies women, focusing on their bodies; Self-objectification can result in adverse psychological consequences, including feelings of body shame and anxiety about appearance.
Social comparison theory (Festinger, 1954)	- People gauge their abilities and qualities by contrasting them with others’; Exposure to idealized imagery can provoke upward social comparisons and unfavorable self-assessments.	- Advertisements featuring idealized images of women can trigger upward social comparisons; These comparisons can lead to increased body dissatisfaction, lowered self-esteem, and negative affect.

As the field of advertising and media psychology continues to evolve, new theoretical perspectives have emerged to account for the changing landscape of media and its impact on self-perception. One such perspective is the postfeminist media culture framework, which has been used to analyze the rise of femvertising and its potential to challenge traditional gender stereotypes (Gill, 2007; McRobbie, 2009). Postfeminist media culture refers to the notion that feminism has achieved its main goals and is no longer necessary, with the emphasis shifting toward individual empowerment and consumer choice (Gill, 2007). In this context, femvertising can be seen as a manifestation of postfeminist media culture, as it often focuses on individual empowerment and the celebration of women’s achievements, while glossing over systemic issues of gender inequality (Gill and Elias, 2014; Johnston and Taylor, 2008).

Scholars of postfeminist media culture assert that femvertising, though seemingly empowering, might paradoxically perpetuate the idea that women’s worth is predominantly linked to their appearance and purchasing power (Johnston and Taylor, 2008; Gill and Elias, 2014). Additionally, the postfeminist focus on individual agency and empowerment could obscure the persisting structural barriers and gender disparities influencing women’s lives (McRobbie, 2009). Table 2 outlines the fundamental principles and consequences of the postfeminist media culture framework, providing insights into how femvertising affects women’s self-perception.

**Table 2:** Postfeminist media culture framework and implications for femvertising.

Framework	Key tenets	Implications for femvertising and self-perception
Postfeminist media culture (Gill, 2007; McRobbie, 2009)	- Feminism is seen as no longer necessary, with a focus on individual empowerment and consumer choice; Media culture emphasizes individual success and personal responsibility.	- Femvertising can be seen as a manifestation of postfeminist media culture, focusing on individual empowerment; Femvertising may reinforce the notion that women’s value is tied to appearance and consumption, obscuring structural inequalities.

By drawing on these theoretical perspectives, this systematic review aims to study the impact of advertising on women’s self-perception. The synthesis of the literature will be informed by these frameworks, allowing for a critical examination of the psychological processes and cultural factors that shape the relationship between advertising and women’s self-perception.

## 2 Methodology

### 2.1 Search strategy

A thorough search of the literature was performed utilizing the following electronic databases: PsycINFO, Communication and Mass Media Complete, Business Source Complete, and Web of Science. The selection of keywords was a systematic process designed to capture the full range of relevant literature. First, we began with broad terms directly related to our research focus: “advertising,” “women,” and “self-perception.” Second, these were expanded to include related concepts and synonyms, such as “marketing,” “female,” “body image,” and “self-esteem.” Then, we conducted pilot searches to refine our keyword list, ensuring comprehensive coverage while maintaining specificity. Finally, terms related to emerging trends in advertising, such as “femvertising,” “social media advertising,” and “influencer marketing” were also included. The full list of search terms used can be found in Table 3.

**Table 3:** Search terms used in the literature search.

Concept	Search terms
Advertising	advertis*, commercial*, marketing, media
Women	women, female*, girl*
Self-perception	self-perception, self-concept, self-image, self-esteem, body image, body satisfaction, self-objectification
Gender roles	gender role*, gender stereotype*, feminis*, femvertis*

The asterisk (*) denotes a wildcard search operator, which allows for the inclusion of variations of the search term (e.g., advertis* includes advertising, advertisement, advertisements, etc.).

The search was confined to peer-reviewed articles published in English from 2000 to 2023. This time frame was chosen for the following reasons: First, it captures significant changes in advertising practices and media consumption patterns in the 21st century; Second, it allows us to track the evolution of research on advertising’s impact on women’s self-perception, including the emergence and development of femvertising. Third, the start date of 2000 ensures we capture foundational studies in the field, while the end date of 2023 allows us to include the most recent research.

Additionally, the reference lists of selected articles and pertinent review papers were scrutinized to uncover supplementary studies.

### 2.2 Inclusion and exclusion criteria

Studies were included in the systematic review if they met the following criteria:

- Empirical investigations exploring the influence of advertising on women’s self-perception, body image, or attitudes toward gender roles.
- Focused on adult women (aged 18 years or older).
- Published in a peer-reviewed journal between 2000 and 2023.
- Written in English.

Studies were excluded if they:

- Focused solely on children or adolescents.
- Did not include a measure of self-perception, body image, or gender role attitudes.
- Were not empirical research, i.e., researches other than quantitative, qualitative, and mixed methods studies.
- Were not published in a peer-reviewed journal or were published outside the specified date range.
- Were not written in English.

### 2.3 Study selection and data extraction

The process of selecting studies adhered to the guidelines outlined in the Preferred Reporting Items for Systematic Reviews and Meta-Analyses (PRISMA) (Moher et al., 2009). The full texts of the remaining articles were then reviewed to determine their eligibility for inclusion. Data from the included studies were extracted using a standardized form. The extracted information included study characteristics (e.g., authors, year, country), sample characteristics (e.g., size, age range), advertising type (e.g., traditional, femvertising), measures of self-perception, body image, or gender role attitudes, and key findings.

### 2.4 Quality assessment

The included studies’ quality was evaluated utilizing the Mixed Methods Appraisal Tool (MMAT) (Hong et al., 2018). This tool is validated for assessing the methodological rigor of qualitative, quantitative, and mixed-methods research. Each study underwent assessment based on five criteria pertinent to its research design, with scores ranging from 0 (indicating no criteria met) to 5 (representing all criteria met).

### 2.5 Data synthesis

Because of the varied research designs, outcome measures, and types of advertising covered in the included studies, a narrative synthesis method was employed (Popay et al., 2006). The study findings were condensed and structured thematically, concentrating on howadvertising and femvertising influence women’s self-perception, body image, and gender role attitudes. The synthesis also highlighted similarities and discrepancies among the studies, and the strength of evidence for each theme was evaluated based on the quantity and quality of the studies.

The results of this synthesis are presented in the following section, structured according to the four objectives we mentioned earlier.

## 3 Results

### 3.1 Study selection

**Figure 1:**
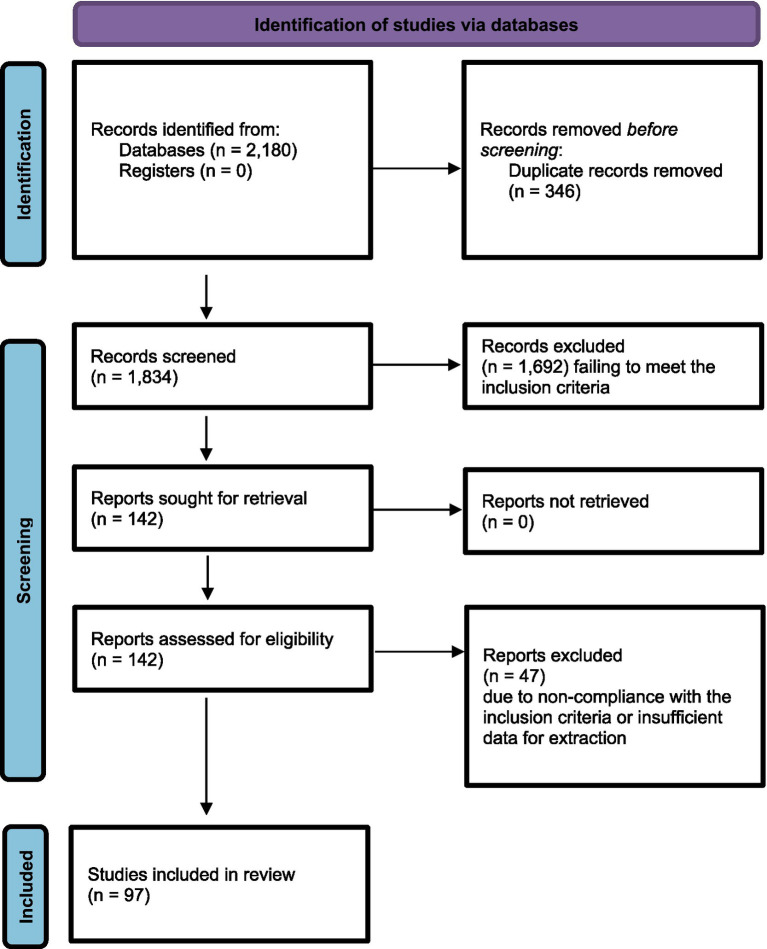
Flow diagram of the research article selection process.

The initial search across databases produced 2,157 articles, and an extra 23 articles were discovered via manual perusal of reference lists. Following the elimination of duplicates, 1,834 articles were subjected to screening based on their titles and abstracts. Among these, 1,692 articles were excluded for failing to meet the inclusion criteria. The full texts of the remaining 142 articles were thoroughly examined, resulting in the exclusion of 47 articles due to non-compliance with the inclusion criteria or insufficient data for extraction. Ultimately, 95 studies met the criteria for inclusion in this systematic review. Below, we present a PRISMA Diagram of our selection process (Figure 1).

### 3.2 Quality assessment

The quality of the included studies was assessed using the MMAT, with scores ranging from 2 to 5. The average quality score was 3.9, indicating that the studies were generally of good quality. Common limitations included cross-sectional designs, reliance on self-report measures, and lack of long-term follow-up assessments.

### 3.3 Impact of advertising on women’s self-perception

#### 3.3.1 Body image and self-objectification

A considerable number of the studies included (*n* = 63) studied the repercussions of advertising on women’s body image and self-objectification. Consistent findings emerged indicating that exposure to idealized depictions of women in advertisements correlated with heightened body dissatisfaction, self-objectification, and internalization of the thin ideal (e.g., Bessenoff, 2006; Harper and Tiggemann, 2008; Tiggemann and McGill, 2004). Grabe et al.'s (2008) meta-analysis revealed a significant link between media exposure and body dissatisfaction, demonstrating a moderate effect size (*d* = 0.41).

Furthermore, several studies underscored the intermediary role of social comparison in the connection between exposure to advertising and adverse body image outcomes (e.g., Bessenoff, 2006; Tiggemann and Polivy, 2010). Women who engaged more frequently in comparisons with the idealized figures portrayed in advertisements reported heightened levels of body dissatisfaction and self-objectification.

Recent studies have further examined the negative impact of advertising on women’s body image and self-objectification. For example, Rodgers et al. (2020) examined the relationships between social media use, psychological factors, and body image concerns among adolescents. Their study found that social media use was associated with lower self-esteem and higher depressive symptoms in both girls and boys. These psychological factors were directly linked to the internalization of appearance ideals and engagement in upward appearance comparisons on social media. The study revealed that the internalization of ideals and appearance comparisons mediated the relationship between social media use and negative outcomes such as body dissatisfaction, dietary restraint, and muscle-building behaviors. Gender differences were observed, with the model explaining a larger proportion of variance in these outcomes for girls compared to boys.

Fardouly et al. (2020) observed that the impact of advertising on body image might be moderated by the type of media platform. Their study revealed that while exposure to idealized images on social media platforms led to greater body dissatisfaction compared to traditional print advertisements, the effect was more persistent for print ads. This suggests that the pervasive nature of digital advertising may have long-lasting effects on women’s self-perception.

Moreover, Tiggemann and Anderberg (2020) found that exposure to thin-ideal advertisements not only increased body dissatisfaction but also led to higher levels of self-objectification and appearance anxiety. Their experimental study demonstrated that even brief exposure to such ads could trigger a self-objectifying state, where women viewed themselves primarily as objects to be evaluated based on appearance.

A study of TikTok beauty content revealed that even brief exposure led to significantly increased appearance shame and anxiety compared to neutral content, with effects emerging immediately after viewing (Seekis and Kennedy, 2023). These findings gain particular significance given that social media has become a primary channel for advertising exposure.

Furthermore, research on female-oriented accounts shows that traditional beauty-focused content may be less effective than previously assumed. Analysis of successful TikTok accounts reveals that authentic connection and emotional engagement are more powerful drivers of audience response than conventional beauty messaging (Du et al., 2022). This suggests that while objectification effects persist, their mechanisms may be evolving in digital contexts.

#### 3.3.2 Self-esteem and mental health

Twenty-eight studies scrutinized the influence of advertising on women’s self-esteem and psychological well-being. Exposure to idealized portrayals of women in advertisements was linked to diminished self-esteem, heightened appearance-related anxiety, and elevated depressive symptomatology (e.g., Birkeland et al., 2005; Durkin and Paxton, 2002; Tiggemann and Kuring, 2004).

The relationship between traditional advertising and women’s self-esteem and mental health continues to be a concern. A longitudinal study by Jarman et al. (2021) found that cumulative exposure to idealized images in advertising over a two-year period was associated with decreased self-esteem and increased depressive symptoms among young adult women. This suggests that the effects of advertising on mental health may be cumulative and long-lasting.

Contemporary research has uncovered more complicated relationships between advertising exposure and psychological well-being. Studies examining digital advertising content demonstrate that impacts on self-esteem operate through multiple pathways. For instance, analysis of TikTok content shows that self-compassion can serve as a protective factor against negative advertising effects on self-esteem (Seekis and Kennedy, 2023). This suggests the relationship between advertising exposure and mental health outcomes is more complex than previously understood.

Selensky and Carels (2021) conducted a study examining the effects of different advertising campaigns on women’s self-esteem, body image, and affect. They compared the impact of body-positive campaigns (Aerie Real and Dove Real Beauty) against traditional thin-ideal promoting campaigns (Victoria’s Secret). The researchers found that women who viewed the Aerie and Dove campaigns reported significantly improved self-esteem and positive affect. Conversely, women exposed to the Victoria’s Secret campaign reported feeling worse about themselves and their bodies. These findings highlight the potential for advertising to either positively or negatively impact women’s self-perception and mental well-being.

Importantly, Selensky and Carels’ study also demonstrated that body-positive advertising campaigns can have immediate positive effects on women’s self-esteem and mood. This suggests that the type of imagery and messaging used in advertising can play a crucial role in shaping women’s self-perception and emotional state. The study underscores the responsibility of advertisers and media to consider the psychological impact of their content on their audience.

#### 3.3.3 Gender role attitudes and stereotypes

Thirty-five studies examined the impact of advertising on women’s gender role attitudes and stereotypes. Exposure to advertisements depicting women in stereotypical roles (e.g., homemakers, sexual objects) was associated with increased endorsement of traditional gender roles and sexist attitudes (e.g., Lavine et al., 1999; MacKay and Covell, 1997; Matthes et al., 2016). Women were underrepresented in professional roles and over represented in decorative and home-related roles in print advertisements (Plakoyiannaki and Zotos, 2009).

Recent research has explored on gender stereotypes in advertising and their impact on women’s self-perception. Kordrostami and Laczniak (2022) examined the portrayal of female power in advertising and identified two distinct dimensions: “power-over” (control over others or the environment) and “power-to” (control over one’s own thoughts, behaviors, and feelings). Their study found that advertisements featuring “power-to” portrayals were more effective in promoting positive self-perception among women, suggesting a potential avenue for more empowering representations in advertising. Åkestam et al. (2021) further expanded our understanding of gender stereotypes in advertising by investigating cross-gender effects. Their study revealed that both women and men react negatively to stereotyped portrayals of other genders in advertising. This negative reaction was mediated by the presumed influence of the ads on others and ad reactance. Interestingly, the study found that stereotyped portrayals led to lower levels of ad attitudes, brand attitudes, and in some cases, purchase intentions across genders compared to non-stereotyped portrayals. These findings suggest that the negative effects of gender-stereotyped advertising are more far-reaching than previously thought, extending to exposure audiences regardless of gender.

### 3.4 Effectiveness of femvertising in promoting positive self-perception

#### 3.4.1 Body image and self-esteem

Seventeen studies probed into the effects of femvertising on women’s body image and self-esteem. Generally, exposure to femvertising campaigns was linked to more favorable body image perceptions and increased self-esteem compared to traditional advertising (e.g., Bissell and Rask, 2010; Kapoor and Munjal, 2019; Taylor et al., 2016). Arendt et al.'s (2016) study revealed that women exposed to a femvertising video reported greater body satisfaction and self-compassion in comparison to those exposed to a traditional advertising video.

Recent studies have provided more nuanced insights into the effectiveness of femvertising in promoting positive body image and self-esteem. Varghese and Kumar (2022) conducted a critical review of femvertising campaigns and found that while many such campaigns aim to promote body positivity, their effectiveness can vary based on perceived authenticity and the specific approach taken.

A study by Couture Bue and Harrison (2019) found that exposure to body-positive advertisements resulted in improved body image and self-esteem compared to traditional thin-ideal advertisements. However, the authors noted that the effectiveness of these campaigns was moderated by participants’ initial levels of internalization of beauty ideals. Women with higher levels of internalized beauty standards showed smaller improvements, suggesting that femvertising may need to be combined with other interventions to be most effective.

The effectiveness of femvertising in promoting positive body image appears highly dependent on authenticity. Recent research identifies specific dimensions that determine whether feminist advertising messages genuinely promote positive self-perception or risk being perceived as “femwashing” (Hainneville et al., 2023). Effectiveness requires alignment across multiple dimensions including transparency, consistency, and genuine diversity rather than superficial empowerment messages.

Digital platforms have created new opportunities and challenges for femvertising’s impact on body image. Studies of social media content show that while traditional femvertising approaches can be effective, engagement and authenticity may be more important than specific message content (Du et al., 2022). Additionally, platform-specific features like algorithmic content selection may influence how femvertising messages are received and interpreted.

#### 3.4.2 Gender role attitudes and empowerment

The impact of femvertising on women’s gender role attitudes and perceived empowerment was examined in 12 studies. Femvertising campaigns were found to challenge traditional gender stereotypes and promote more egalitarian attitudes (e.g., Akestam et al., 2017; Drake, 2017). A content analysis by Feng et al. (2019) revealed that femvertisements often depicted women in non-traditional roles and emphasized themes of empowerment and self-confidence.

However, some studies also highlighted potential limitations of femvertising, such as the risk of reinforcing postfeminist notions of individual empowerment over collective action (e.g., Gill and Elias, 2014; Johnston and Taylor, 2008) and the emphasis on consumption as a means of achieving empowerment (e.g., Abitbol and Sternadori, 2016; Vredenburg et al., 2020).

The impact of femvertising on gender role attitudes and empowerment has been a subject of recent scholarly attention. Windels et al. (2020) analyzed femvertising campaigns and identified several common discourses of female empowerment, including individual choice, inner beauty, and collective action. However, they also noted that many campaigns still operate within a postfeminist framework that emphasizes individual empowerment over systemic change.

Åkestam et al. (2017) found that femvertising campaigns that challenge gender stereotypes can lead to more egalitarian attitudes among both women and men. Their study revealed that exposure to counter-stereotypical portrayals of women in advertising was associated with decreased endorsement of traditional gender roles and increased support for gender equality.

### 3.5 Factors influencing the impact of advertising on women’s self-perception

Several factors were identified as potential moderators of the relationship between advertising exposure and women’s self-perception. These included individual differences in internalization of the thin ideal (e.g., Dittmar and Howard, 2004), self-objectification (e.g., Harper and Tiggemann, 2008), and feminist beliefs (e.g., Burn et al., 2000). Additionally, the perceived authenticity and credibility of femvertising messages were found to influence their effectiveness in promoting positive self-perception (e.g., Abitbol and Sternadori, 2016; Drake, 2017).

Previous research has identified several key factors that moderate the impact of advertising on women’s self-perception. Fardouly et al. (2020) found that media literacy skills play a crucial role in mitigating the negative effects of idealized images on body image. Their study showed that women with higher levels of critical media literacy were less likely to engage in appearance comparisons and reported higher body satisfaction after exposure to traditional advertisements.

The role of social media in shaping the impact of advertising has gained increased attention. Garcia et al. (2022) found that daily Instagram use was positively associated with increased state self-objectification among college-aged women. Their study, which utilized a daily diary method, revealed that higher Instagram use on a given day was related to more self-objectifying thoughts and feelings on that same day. Moreover, increased daily Instagram use was linked to lower life satisfaction and higher negative mood. These findings suggest that the frequent exposure to visual content on social media platforms, which often includes idealized images and advertisements, may exacerbate the negative effects of traditional advertising on women’s self-perception and well-being. The study highlights the importance of considering the cumulative and immediate impacts of social media exposure when examining the broader effects of advertising on women’s self-objectification and mental health.

Cultural factors also play a significant role in shaping women’s responses to advertising. Terlutter et al. (2022) conducted a cross-cultural study comparing reactions to potentially offensive nudity advertising in China and Austria. They found that Chinese consumers perceived higher levels of offensiveness in nudity ads compared to Austrian consumers. Interestingly, the study revealed that same-ethnicity models in nudity advertising led to higher perceived offensiveness and more negative advertising outcomes in China, whereas in Austria, same-ethnicity models resulted in lower perceived offensiveness and more positive outcomes. This suggests that cultural norms and values, such as collectivism and restraint in China versus individualism and indulgence in Austria, significantly influence how women perceive and respond to potentially offensive advertising content.

Recent studies have revealed additional complexity in the factors moderating advertising’s impact. Research on digital platforms indicates that content authenticity and emotional connection may be more important moderating factors than traditional variables like exposure frequency or message type (Du et al., 2022). Platform characteristics also appear to play a crucial role, with different digital environments creating distinct contexts for message reception and interpretation.

Furthermore, consumer sophistication in evaluating feminist advertising claims has emerged as an important moderating factor. Hainneville et al. (2023) demonstrate that consumers apply complex frameworks to evaluate advertising authenticity, considering multiple dimensions simultaneously. This suggests that individual differences in media literacy and feminist consciousness may significantly moderate advertising impacts. Additionally, cultural context appears increasingly important as a moderating factor, with responses to both traditional and feminist advertising varying significantly across different cultural settings.

### 3.6 Digital transformation of advertising effects

Digital technologies have fundamentally transformed how advertising affects people’s self-perception and psychological well-being. While traditional media offered scheduled advertising encounters, social media platforms now provide continuous exposure to commercial content (Merino et al., 2024).

Research shows that digital platforms intensify advertising exposure in several ways. On Instagram, users now spend significantly more time engaging with content compared to other websites and social media channels due to its video-based features and detailed information (Shahbaznezhad et al., 2022). According to Alfonso-Fuertes et al. (2023), a study of daily Instagram use found that higher usage was associated with increased state self-objectification and lower body image satisfaction and self-esteem among young adults aged between 18 years and 40 years old.

Social media influencers have emerged as powerful advertising channels, with their (Lee and Lee, 2022) effectiveness often depending on parasocial interactions and perceived similarity. Research by Hudders and De Jans (2022) found that women tend to feel more similar to female influencers compared to male influencers, leading to stronger parasocial interactions and greater message effectiveness. Interestingly, their study revealed that men showed no significant differences in their responses to male versus female influencers.

Digital platforms have also transformed social comparison processes. Fioravanti et al. (2023) conducted an intensive longitudinal study examining how exposure to different types of Instagram content affects young women. Their research demonstrated that viewing body-positive content led to higher rates of positive mood and body satisfaction, while exposure to fitspiration content was associated with increased negative mood and appearance comparison.

Platform-specific features create distinct advertising environments. Merino et al. (2024) note that the emphasis on physical measurements and appearance in digital advertising can significantly impact psychological well-being, particularly when amplified by algorithmic content selection. Their research highlights how cultural norms and gender factors intersect with digital advertising to influence body image perceptions.

Looking forward, emerging technologies continue to reshape advertising’s psychological impact. As Merino et al. (2024) discuss, virtual reality and artificial intelligence-driven content raise new questions about personalization’s effects on mental health and body image. These developments require attention from researchers developing new methodologies to study platform-specific effects, healthcare professionals understanding different advertising approaches’ impact on psychological well-being, and policymakers establishing frameworks that protect users while allowing innovation.

The evidence suggests that understanding digital advertising’s impact requires consideration of platform features, user characteristics, and content types. This understanding becomes increasingly important as social media platforms continue to evolve and influence how people perceive themselves and others.

## 4 Discussion

### 4.1 Summary of findings

This systematic review provides a comprehensive synthesis of research examining advertising’s psychological impact on women’s self-perception. Our analysis of 95 peer-reviewed studies reveals several key findings. Traditional advertising continues to have detrimental effects on women’s psychological well-being, manifesting through increased body dissatisfaction, self-objectification, decreased self-esteem, and reinforcement of gender stereotypes. These effects appear particularly pronounced in digital contexts, where exposure is more frequent and pervasive.

Femvertising shows promise but faces important limitations. While it can positively affect body image and self-esteem when perceived as authentic, there is a risk of superficial empowerment messaging that fails to address systemic issues. The effectiveness of femvertising appears contingent on perceived authenticity and individual viewer characteristics, with more sophisticated consumers able to distinguish between genuine and performative feminist messaging.

The digital transformation of advertising has fundamentally altered how women encounter and process advertising messages. Our findings indicate that digital platforms intensify advertising exposure while creating new mechanisms of influence through interactivity, algorithmic content selection, and community engagement (Du et al., 2022; Seekis and Kennedy, 2023). These technological changes have accelerated the temporal dynamics of advertising effects while creating more complex pathways of influence.

### 4.2 Theoretical implications

Our findings extend existing theoretical frameworks in several important ways while suggesting the need for theoretical evolution to account for contemporary advertising contexts. The synthesis of 95 studies provides robust support for key theoretical perspectives while revealing new dimensions that require theoretical consideration.

Objectification theory (Fredrickson and Roberts, 1997) gains substantial support and extension through our findings. The theory’s core premise that women internalize an observer’s perspective of their physical selves continues to explain advertising effects, but with new dimensions in digital contexts. Our synthesis reveals that self-objectification processes operate differently across advertising platforms, with social media creating unique conditions for self-objectification through both consumption and creation of content (Garcia et al., 2022). The interactive nature of digital platforms appears to intensify self-objectification processes, as women simultaneously serve as both observers and observed (Tiggemann and Anderberg, 2020). Recent research on TikTok beauty content demonstrates how even brief exposure can trigger immediate self-objectification effects, suggesting that digital platforms may accelerate the processes described in objectification theory (Seekis and Kennedy, 2023).

Social comparison theory (Festinger, 1954) requires significant updating for contemporary advertising contexts. While traditional upward social comparison processes remain relevant, digital platforms have transformed how these comparisons occur. Our findings align with recent research showing that social media creates continuous opportunities for appearance-related comparisons, with both traditional advertising and user-generated content serving as comparison targets (Fardouly et al., 2020). The immediacy and interactivity of digital platforms appear to intensify social comparison effects, while also introducing new moderating factors such as perceived authenticity and platform-specific features (Rodgers et al., 2020). Furthermore, the distinction between advertising and non-advertising content has become increasingly blurred on social media, creating new theoretical challenges for understanding comparison processes (Du et al., 2022).

The postfeminist media culture framework (Gill, 2007; McRobbie, 2009) gains new relevance in analyzing contemporary advertising’s impact on women’s self-perception. Our synthesis reveals complex tensions between individual and collective empowerment messages, particularly in how feminist advertising navigates commercial imperatives and social change goals. Recent research has identified six key dimensions that shape perceptions of authentic feminist advertising: transparency, consistency, identification, diversity, respect, and challenging stereotypes (Hainneville et al., 2023). These dimensions suggest the need for theoretical models that can account for both traditional feminist critiques and emerging forms of resistance and reinterpretation.

These theoretical extensions point toward the need for an integrated framework that accounts for several key factors. First, such a framework must address multi-platform advertising exposure, recognizing that women encounter advertising messages across various media contexts simultaneously (Windels et al., 2020). Second, it must account for the interaction between traditional and digital effects, including how different forms of advertising may reinforce or contradict each other (Terlutter et al., 2022). Third, the role of authenticity in determining advertising impact must be theoretically centered, particularly in understanding responses to feminist advertising messages. Finally, individual differences in processing and response need theoretical attention, including factors such as media literacy, feminist consciousness, and cultural context (Varghese and Kumar, 2022).

Recent research also suggests the need to theorize the role of consumer sophistication in evaluating advertising claims. Studies indicate that consumers have developed increasingly nuanced frameworks for assessing advertising authenticity, particularly regarding feminist messages (Champlin et al., 2019). This sophistication appears to moderate advertising effects, with more media-literate consumers showing different response patterns to both traditional and feminist advertising (Kordrostami and Laczniak, 2022).

The rapid evolution of digital advertising platforms necessitates ongoing theoretical development. Future theoretical work should focus on integrating insights from traditional advertising theories with new understanding of digital media effects. This includes examining how platform-specific features may moderate or mediate advertising impacts, how algorithmic content selection shapes exposure patterns, and how different forms of advertising interaction affect women’s self-perception.

### 4.3 Practical implications

The findings of this systematic review have significant implications for multiple stakeholders involved in advertising creation, regulation, and consumer protection. For advertising practitioners, our analysis suggests the need for a fundamental shift in approach that considers both traditional and digital advertising effects. Advertisers must recognize that the cumulative impact of exposure across multiple platforms may intensify psychological effects beyond what was previously understood. As demonstrated by recent research (Du et al., 2022), authentic connection and emotional engagement have become more important than conventional beauty messaging in driving audience response.

The digital transformation of advertising necessitates new approaches to content creation and distribution. Advertisers should consider how algorithmic content selection might amplify certain messages and potentially exacerbate negative psychological effects. The findings of Seekis and Kennedy (2023) regarding immediate psychological impacts from brief social media exposure suggest that advertisers need to carefully consider the frequency and intensity of their digital advertising campaigns.

For brands pursuing feminist advertising approaches, our review highlights the critical importance of authenticity. As Hainneville et al. (2023) demonstrate, consumers have developed sophisticated frameworks for evaluating feminist advertising claims. Brands must ensure alignment across multiple dimensions including transparency, consistency, identification, diversity, respect, and challenging stereotypes. This requires moving beyond superficial empowerment messages to demonstrate genuine commitment to gender equality through both messaging and organizational practices.

Mental health professionals and healthcare providers should be aware of how different advertising formats may affect their clients’ psychological well-being. The findings of Garcia et al. (2022) regarding daily social media use and self-objectification suggest the need to consider platform-specific effects when developing interventions. Treatment approaches may need to address both traditional advertising exposure and digital media consumption patterns.

For media literacy educators and consumer advocates, our findings indicate the need for updated approaches that address both traditional and emerging advertising formats. Programs should help individuals understand how different platforms may affect self-perception and develop critical evaluation skills for both conventional and digital advertising content. Special attention should be paid to the role of algorithmic content selection and the blurring boundaries between advertising and authentic content.

Policymakers and regulatory bodies face new challenges in protecting consumers from potentially harmful advertising effects. Traditional regulatory frameworks may need updating to address the rapid pace of digital advertising evolution and the unique features of different platforms. Particular attention should be paid to the cumulative effects of cross-platform exposure and the potential amplification of messages through algorithmic content selection.

Platform developers and technology companies should consider how their features and algorithms might affect the psychological impact of advertising content. The findings regarding platform-specific effects suggest the need for design approaches that minimize potential negative impacts while maintaining engagement. This might include developing better content moderation systems and more transparent algorithmic selection processes.

For researchers and academics, our findings suggest the need for new methodological approaches to studying advertising effects. Future research should employ methods capable of capturing cross-platform exposure and measuring cumulative effects over time. This might include developing new measurement tools and approaches for studying advertising impact in digital environments.

### 4.4 Limitations and future directions

This systematic review has several important limitations that should be considered when interpreting its findings and planning future research. The limitations span methodological, theoretical, and practical dimensions, each suggesting specific directions for future investigation.

A primary methodological limitation of the current literature is the predominance of Western samples, particularly from North America and Western Europe. While some studies have examined advertising effects in Asian contexts (e.g., Terlutter et al., 2022), there remains limited understanding of how advertising impacts women’s self-perception across diverse cultural contexts. This geographic bias limits the generalizability of findings and leaves gaps in our understanding of cultural moderators.

The reliance on cross-sectional designs in many studies (*n* = 63 of 95 reviewed studies) limits our ability to draw causal conclusions about advertising’s long-term effects. While experimental studies have demonstrated immediate impacts of advertising exposure (e.g., Tiggemann and Anderberg, 2020), the cumulative effects of sustained exposure across multiple platforms remain poorly understood. Additionally, most studies rely heavily on self-report measures, which may be subject to social desirability bias and recall errors.

Another significant limitation is the rapidly evolving nature of digital advertising platforms, which poses challenges for research currency. Studies examining specific platform effects may become outdated as new features and formats emerge. The current literature also shows limited integration of advertising exposure across multiple platforms, potentially underestimating cumulative effects.

Sample characteristics present additional limitations. Many studies focus on college-aged women, limiting our understanding of advertising effects across different age groups. The literature also shows limited examination of intersectional factors such as race, socioeconomic status, and sexual orientation. Furthermore, measurement approaches vary considerably across studies, making direct comparisons challenging and meta-analytic integration difficult.

Based on these limitations, several priority areas emerge for future research. Longitudinal research should examine the developmental trajectory of advertising effects across the lifespan, cumulative impacts of exposure across multiple platforms, long-term effectiveness of femvertising approaches, and changes in self-perception over time in response to evolving advertising landscapes. Such studies would provide crucial insights into how advertising effects develop and persist over time.

Cultural and intersectional analysis represents another critical direction for future research. Studies should expand to include more diverse cultural contexts, particularly non-Western settings, and employ intersectional approaches examining multiple identity factors. Cross-cultural comparisons of femvertising effectiveness and investigation of cultural moderators would enhance our understanding of how advertising effects vary across different populations.

Research on digital platform dynamics should prioritize understanding platform-specific effects on self-perception and the interaction between traditional and digital advertising formats. The role of algorithmic content selection in shaping exposure patterns and the impact of user-generated content and influencer marketing require particular attention as these features increasingly define the modern advertising landscape.

Future research should also focus on measurement development, creating standardized measures of advertising exposure across platforms and validated tools for assessing femvertising authenticity. Methods for measuring cumulative advertising effects and improving ecological validity in advertising exposure assessment are particularly needed to advance the field.

Investigation of individual differences represents another crucial research direction. Studies should examine protective factors against negative advertising effects, the role of media literacy in moderating advertising impact, and the influence of feminist consciousness on advertising interpretation. Understanding age-related differences in advertising susceptibility and the impact of prior mental health conditions would help identify vulnerable populations and develop targeted interventions.

The development and evaluation of interventions should focus on designing and testing media literacy programs, developing platform-specific protective strategies, and creating evidence-based guidelines for authentic feminist advertising. Evaluation of existing intervention approaches would help identify effective strategies for promoting positive self-perception in the face of pervasive advertising exposure.

Methodological advances should incorporate mixed-method designs combining quantitative and qualitative approaches, ecological momentary assessment of advertising exposure, and neuroimaging studies of advertising processing. Big data approaches to tracking advertising exposure and natural experiments examining platform changes would provide new insights into advertising effects.

Theoretical development should focus on integrating existing frameworks for digital contexts and developing new theories addressing multi-platform effects. Expanded understanding of authenticity in feminist advertising and models of cumulative advertising impact would enhance our theoretical foundation for understanding advertising effects.

These future directions should be pursued with attention to methodological rigor and practical applicability. Researchers should prioritize using diverse and representative samples, employing multiple measurement approaches, and considering practical implications for advertisers and policymakers while maintaining awareness of rapidly evolving media landscapes.

## 5 Conclusion

This systematic review advances our understanding of advertising’s psychological impact on women’s self-perception in several key ways. While previous scholars focused primarily on body image concerns, our review provides a more comprehensive examination of advertising’s psychological effects across multiple domains. Our findings both confirm and extend previous research while identifying emerging trends in the contemporary media landscape.

Our synthesis reveals three significant advances in understanding. First, while earlier studies found moderate effects of traditional advertising on body dissatisfaction, our review suggests these effects may be intensifying in digital contexts, particularly through social media advertising. Second, our analysis of femvertising extends beyond previous content analyses by identifying the crucial role of perceived authenticity in determining psychological outcomes. Third, our findings highlight how the simultaneous exposure to multiple advertising formats creates more complex effects than previously documented in studies examining traditional advertising alone.

Several key implications emerge from this analysis. The digital transformation of advertising has created both new challenges and opportunities that require updated theoretical frameworks and practical approaches. While traditional advertising continues to pose risks to psychological well-being, emerging forms of feminist advertising offer potential benefits when executed authentically. However, the effectiveness of these approaches depends heavily on perceived authenticity and individual viewer characteristics.

Looking forward, this review identifies critical areas for future research. Longitudinal studies are needed to understand the cumulative impact of advertising exposure across platforms over time. Cross-cultural research should examine how cultural factors moderate both traditional and emerging advertising effects. Additionally, research must continue to evolve alongside the rapidly changing media landscape, maintaining focus on both theoretical advancement and practical application.

In conclusion, while our findings confirm many previous concerns about advertising’s impact on women’s self-perception, they also reveal new dynamics and potential opportunities for positive change. The field must continue to adapt to rapid changes in the advertising landscape while developing effective interventions to promote positive self-perception among women.

## Data Availability

The original contributions presented in the study are included in the article/supplementary material, further inquiries can be directed to the corresponding author/s.
